# Combined PD-L1/CD8 immune phenotyping in breast cancer: exploratory associations across molecular subtypes and insights into possible exhaustion-related immune dysfunction

**DOI:** 10.3389/fmed.2026.1896491

**Published:** 2026-08-13

**Authors:** Burcu Sanal Yilmaz, Afife Uguz, Sibel Acat, Merve Tuna Kaptan, Hatice Deniz, Ceyhan Ugurluoglu, Zeliha Esin Celik

**Affiliations:** 1 Department of Pathology, Karamanoglu Mehmetbey University, Karaman, Türkiye; 2 Selcuk Universitesi Tip Fakultesi, Konya, Türkiye

**Keywords:** adaptive immune resistance, breast cancer, CD8, immune phenotype, PD-L1, t cell exhaustion, tumor microenvironment, tumor-infiltrating lymphocytes

## Abstract

**Background:**

Programmed death-ligand 1 (PD-L1) expression and CD8+ tumor-infiltrating lymphocyte (TIL) density are established biomarkers of tumor–immune interaction in breast cancer; however, the biological and prognostic significance of their combined assessment across molecular subtypes remains incompletely defined. This study aimed to characterize combined PD-L1/CD8 immune phenotypes, evaluate their clinicopathological associations, and explore their relationship with survival outcomes in a molecularly heterogeneous breast cancer cohort.

**Methods:**

This multicenter retrospective study included 137 patients with invasive breast carcinoma from two institutions. PD-L1 expression was evaluated separately in tumor cells (TCs) and tumor-infiltrating lymphocytes (TILs) by immunohistochemistry. CD8+ TIL density was also assessed immunohistochemically, and tumors were classified as CD8-high or CD8-low using a predefined threshold of ≥50%. Based on combined PD-L1 and CD8 status, cases were categorized into four immune phenotypes: Type I (PD-L1+/CD8-high), Type II (PD-L1+/CD8-low), Type III (PD-L1–/CD8-high), and Type IV (PD-L1–/CD8-low). Associations between immune phenotypes, clinicopathological characteristics, and survival outcomes were analyzed using appropriate statistical methods.

**Results:**

PD-L1 positivity was observed in 62 of 137 tumors (45.3%) and was significantly associated with higher histological grade (*p* = 0.020), but not with pathological tumor stage, clinical stage group, or lymph node status. A significant positive correlation was identified between PD-L1 expression in the immune-cell compartment and CD8+ TIL density (Spearman ρ = 0.453, *p* < 0.001), whereas no correlation was observed between tumor cell PD-L1 expression and CD8+ TIL density (ρ = −0.021, *p* = 0.812). In a hypothesis-generating subgroup comparison limited by small cell counts, CD8-high status was more frequent in Luminal B than in triple-negative breast cancer (36.0 vs. 13.7%; *p* = 0.036). The most common immune phenotype was Type IV (43.1%), whereas Type I accounted for 9.5% of cases. In exploratory analysis, patients with the Type I phenotype had the highest mortality rate (23.1%) and the shortest median follow-up-based survival estimate (37 months) among the four groups (log-rank *p* = 0.014); given the small number of events (11 deaths overall, 3 within the Type I subgroup) and the use of follow-up duration with cross-sectional mortality rather than fully annotated time-to-event data, these findings should be interpreted as exploratory associations rather than definitive prognostic evidence.

**Conclusions:**

Combined PD-L1/CD8 immune phenotyping identifies biologically distinct immune microenvironmental states in breast cancer beyond conventional molecular subtype classification. While PD-L1 positivity was associated with high histological grade and immune-cell infiltration, the PD-L1+/CD8-high phenotype demonstrated the least favorable survival profile in this predominantly hormone receptor-positive cohort on unadjusted analysis, an association that was attenuated to borderline significance after adjustment for baseline metastatic burden. These findings support the concept that concurrent PD-L1 expression and abundant CD8+ T-cell infiltration may not invariably reflect effective antitumor immunity and may instead be consistent with an exhausted or functionally impaired immune state. If validated in larger cohorts with direct assessment of T-cell exhaustion markers, combined PD-L1/CD8 phenotyping may provide clinically relevant insights for immune stratification and patient selection in immunotherapy-oriented approaches.

## 1 Introduction

Breast cancer is the most frequently diagnosed malignancy among women worldwide and a leading cause of cancer-related mortality (1). It encompasses biologically distinct molecular subtypes Luminal A, Luminal B, HER2-enriched, and triple-negative breast cancer (TNBC) that differ in carcinogenesis, tumor microenvironment composition, and prognosis (2). TNBC, defined by the absence of estrogen receptor (ER), progesterone receptor (PR), and HER2 expression, is characterized by aggressive clinical behavior, high relapse rates, and, until recently, the absence of validated targeted therapies (3).

The tumor immune microenvironment (TME) is a critical determinant of breast cancer biology and therapeutic response. CD8+ cytotoxic T lymphocytes represent the principal effectors of antitumor immunity, and their intratumoral density commonly referred to as tumor-infiltrating lymphocytes (TILs) has been established as a favorable prognostic factor, most consistently in TNBC and HER2-positive subtypes (4, 5). Beyond CD8 immunohistochemistry, overall stromal tumor-infiltrating lymphocyte (TIL) density assessed on routine hematoxylin and eosin-stained sections has been standardized by the International TILs Working Group (IWG) and robustly validated as an independent favorable prognostic and predictive biomarker, particularly in TNBC and HER2-positive disease (6). Because stromal TIL density is a more accessible, pan-immune-cell readout than lineage-specific IHC, it provides a complementary screening-level indicator of cytotoxic infiltration in settings where CD8-specific staining is unavailable, and is therefore evaluated alongside PD-L1 and CD8 in the present study. Despite the presence of cytotoxic immune infiltration, tumors evade immune destruction by upregulating immune checkpoint molecules. The programmed cell death ligand 1 (PD-L1) / programmed cell death 1 (PD-1) axis is among the most clinically significant of these mechanisms, suppressing CD8+ T cell effector function and promoting T cell exhaustion within the TME (7, 8).

The relationship between PD-L1 expression and CD8+ TIL infiltration is not one of simple antagonism. PD-L1 upregulation on tumor cells is frequently induced by interferon-gamma (IFN-γ) secreted by infiltrating CD8+ T cells themselves, through the JAK/STAT1 and PI3K/AKT signaling pathways — a phenomenon termed adaptive immune resistance (9, 10). Under this model, PD-L1 positivity may paradoxically indicate an immunologically active microenvironment, and its co-occurrence with high CD8+ TIL density may identify tumors most susceptible to checkpoint blockade. Supporting this paradigm, Wang et al. demonstrated that combined PD-L1-positive/CD8-high status was the sole independent prognostic parameter in basal-like breast cancer (RFS hazard ratio 0.12; p = 0.010) (11). Similarly, Hou et al. reported that both PD-L1 expression and high intratumoral CD8+ density independently predicted better overall survival in HER2-positive breast carcinoma (12), and Çetin et al. identified high CD8+ infiltration as an independent predictor of pathological complete response to neoadjuvant therapy (OR 4.22; *p* = 0.047) (13). However, the prognostic meaning of the combined PD-L1+/CD8-high state is not uniform across all breast cancer subtypes. Emerging evidence indicates that high CD8+ T cell infiltration does not invariably reflect effective antitumor immunity; in hormone receptor-positive (HR+) disease in particular, abundant CD8+ T cells may instead represent an exhausted population characterized by progressive loss of effector function, sustained inhibitory receptor co-expression (PD-1, CD39, TIM-3), and epigenetically fixed dysfunction (14, 15). In this exhaustion-driven state, PD-L1 upregulation persists as a consequence of chronic IFN-γ stimulation rather than as a marker of active immune engagement, creating an immunohistochemical signature of adaptive resistance in the absence of effective cytotoxic surveillance (14). This distinction — between immune activation and immune exhaustion — has direct consequences for biomarker interpretation and patient selection in checkpoint blockade strategies, yet remains inadequately characterized across the full spectrum of breast cancer molecular subtypes and clinical stages.

Clinically, atezolizumab and pembrolizumab have been incorporated into the management of metastatic and early high-risk TNBC, respectively, based on the IMpassion130 and KEYNOTE-522 trials (16, 17). Nevertheless, PD-L1 as a single biomarker demonstrates imperfect predictive performance: a proportion of PD-L1-negative tumors derive benefit from checkpoint inhibition, while many PD-L1-positive tumors do not respond (18). Combined assessment of PD-L1 with CD8+ TIL density has therefore been proposed as a more informative biomarker strategy. However, systematic characterization of these markers across the full spectrum of breast cancer clinical stages and molecular subtypes remains limited, particularly from multicenter archival cohorts.

The present study aimed to: (i) determine the prevalence of PD-L1 expression and CD8+ TIL infiltration across molecular subtypes (TNBC vs. hormone receptor-positive [HR+]) and clinical stages (early, locally advanced, and advanced); (ii) evaluate their correlation and characterize the combined PD-L1/CD8 immune phenotype distribution across clinicopathological categories; (iii) assess survival outcomes by combined immune phenotype and investigate whether the prognostic signal of the PD-L1+/CD8-high phenotype reflects adaptive immune activation or exhaustion-driven immune dysfunction in a molecularly heterogeneous cohort; and (iv) assess associations with key clinicopathological parameters.

## 2 Materials and methods

### 2.1 Study design and ethics

This retrospective, multicenter observational study was conducted at the Department of Pathology, Karamanoglu Mehmetbey University Faculty of Medicine at Karaman Training and Research Hospital and the Department of Pathology, Selcuk University Faculty of Medicine.

The study protocol was conducted in accordance with the principles of the Declaration of Helsinki and was approved by the Clinical Research Ethics Committee of Karamanoglu Mehmetbey University Faculty of Medicine *(date: 09.04.2025, decision no: 18-2025/22)*. As the study had a retrospective design, the requirement for written informed consent was deemed exempt by the ethics committee.

All patient data were anonymized, and full compliance with confidentiality and data security principles was ensured.

### 2.2 Patient selection

FFPE tumor tissue blocks from patients with histologically confirmed invasive breast carcinoma were retrieved from the pathology archives of two centers (January 2009–December 2025).

Inclusion criteria were:

(i) Histologically confirmed invasive breast carcinoma;
(ii) Adequate FFPE tumor tissue (viable tumor area ≥5 mm^2^, ≥100 invasive carcinoma cells);
(iii) documented ER, PR, and HER2 status; and
(iv) Complete core clinicopathological data.

Cases were excluded if patients had received neoadjuvant systemic therapy prior to tissue sampling, if tissue quality was insufficient for reliable immunohistochemical evaluation, or if HER2 equivocal (IHC 2+) status could not be resolved by fluorescence *in situ* hybridization (FISH). A final cohort of 137 patients was included in the study.

### 2.3 Clinicopathological classification

Histological type and grade were assessed per the 2019 WHO Classification of Tumors of the Breast and the Nottingham Grading System, respectively. Clinical stage was assigned according to the AJCC TNM Classification, 8th Edition, and categorized as early stage (Stages I–II), locally advanced stage (Stage III), and advanced/metastatic stage (Stage IV).

ER and PR positivity was defined as nuclear staining in ≥1% of tumor cells per 2020 ASCO/CAP criteria. HER2 status was determined per the 2018 ASCO/CAP guidelines; IHC 2+ cases were reflexed to dual-probe FISH for amplification assessment.

For primary analysis, cases were grouped as TNBC (ER–, PR–, HER2–) or hormone receptor-positive (HR+) (ER+ and/or PR+, any HER2 status). This binary grouping reflects the fundamental distinction in immunological activity between these subtypes in the context of checkpoint biology.

### 2.4 Immunohistochemistry

All staining was performed on 4-μm FFPE sections using the Ventana BenchMark ULTRA automated immunostainer (Ventana Medical Systems, Tucson, AZ, USA) with the OptiView DAB IHC Detection Kit, ensuring identical protocols across both centers.

PD-L1 was assessed using the VENTANA PD-L1 (SP263) assay (Ventana; cat. no. 790-4905), a rabbit monoclonal antibody with established analytical concordance to the 22C3 and 28-8 assays (19). Antigen retrieval was performed in Cell Conditioning 1 (CC1; pH 8.0) buffer at 95–100 °C for 64 min. Placental syncytiotrophoblast served as the positive tissue control.

**Figure 1:**
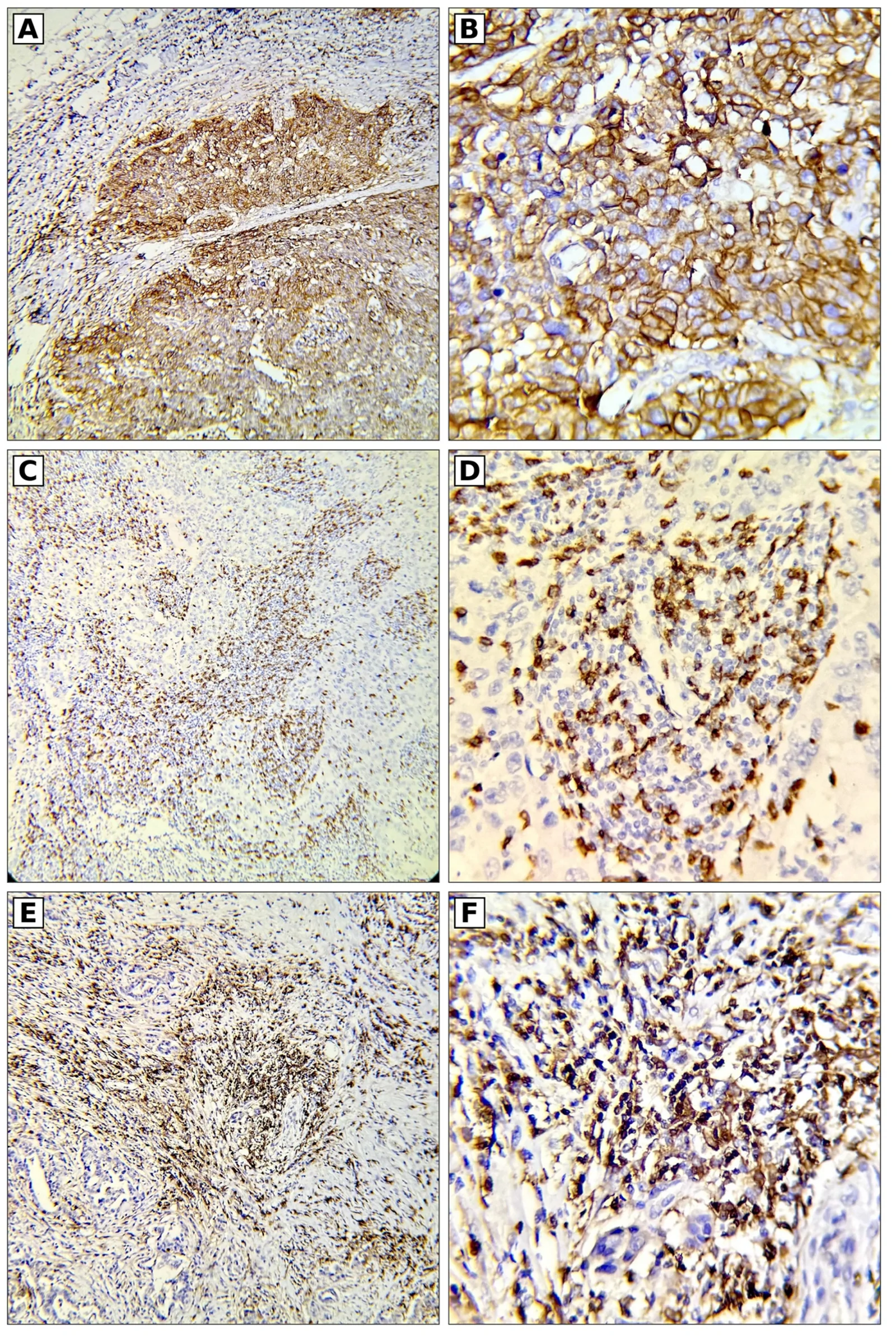
Immunohistochemical expression of PD-L1 and CD8 in breast carcinoma tissue. **(A)** PD-L1 expression in tumor cells, × 100. **(B)** PD-L1 expression in tumor cells, × 400. **(C)** CD8+ tumor-infiltrating lymphocytes, × 100. **(D)** CD8+ tumor-infiltrating lymphocytes, × 400. **(E)** PD-L1 expression in tumor-infiltrating lymphocytes, × 100. **(F)** PD-L1 expression in tumor-infiltrating lymphocytes, × 400.

CD8 was evaluated using clone C8/144B 790-4460 (Ventana/Roche). Antigen retrieval was performed in CC1 (pH 6.0) at 95 °C for 32 min. Tonsil tissue served as the positive control. Both antibodies were incubated for 16 min at 36 °C and visualized with DAB chromogen and hematoxylin counterstain (Figure 1).

### 2.5 Immunohistochemical scoring

All slides were independently evaluated by two pathologists blinded to clinical data. Discordant cases were resolved by consensus review at a multiheaded microscope. Interobserver agreement was quantified using Cohen's kappa (κ); agreement was substantial for both PD-L1 (κ = 0.78) and CD8+ TIL scoring (κ = 0.81).

#### 2.5.1 PD-L1 Scoring

PD-L1 expression was assessed separately in tumor cells (TC) and tumor-infiltrating immune cells (TIL) at × 20 magnification by scanning the entire viable tumor area.

##### 2.5.1.1 PD-L1 expression on tumor cells (PD-L1 TC)

The proportion of viable invasive carcinoma cells showing membranous staining of any intensity was determined at × 20 magnification across the full tumor section. PD-L1 TC positivity was defined as membranous staining in ≥1% of viable tumor cells (PD-L1+ TC). Unless otherwise specified as PD-L1 TIL status, the unqualified term “PD-L1 positivity” used throughout the Results, Tables, and Discussion refers to this tumor cell (TC) positivity criterion (≥1%).

This ≥1% threshold was adopted *a priori* as the standard clinical positivity criterion for PD-L1 TC staining, consistent with established scoring conventions and regulatory-approved diagnostic assays (18, 19), rather than derived from the present dataset. As a *post hoc* concordance check, ROC analysis using overall survival as the outcome identified an independently Youden-optimal threshold of 1% (AUC = 0.563), confirming agreement between the predefined clinical cutoff and the data-driven optimum.

##### 2.5.1.2 PD-L1 expression on TILs (PD-L1 TIL)

PD-L1 expression was evaluated on tumor-infiltrating immune cells by assessing the proportion of the tumor area occupied by PD-L1-positive TILs of any staining intensity at × 20 magnification. The tumor area encompassed viable tumor cells, associated intratumoral stroma, and contiguous peritumoral stroma. PD-L1 positivity in TILs was determined by the percentage of PD-L1-positive TILs relative to the total TIL count within this area. A ≥50% (majority) threshold was applied *a priori* to define PD-L1 TIL-high status, consistent with the conventional biological interpretation of TIL positivity as representing a majority of the infiltrate; cases were classified as PD-L1 TIL-high (≥50%) or PD-L1 TIL-low (< 50%) accordingly. As a *post hoc* concordance check, ROC analysis using overall survival as the outcome identified an independently Youden-optimal cutoff of 50% (AUC = 0.589, sensitivity 0.682, specificity 0.549, Youden index 0.231), confirming agreement between the predefined majority threshold and the data-driven optimum.

#### 2.5.2 TIL and CD8+ T cell assessment

##### 2.5.2.1 Stromal TIL density

The proportion of the tumor-associated stromal area occupied by mononuclear inflammatory cells (lymphocytes and plasma cells) was estimated at × 20 magnification by scanning the entire tumor section, in accordance with the International TILs Working Group 2014 recommendations (6). TIL density was expressed as a percentage of stromal area and used as a continuous variable in correlation analyses.

##### 2.5.2.2 CD8+ TIL density

A ≥50% (majority) threshold was applied *a priori* to define CD8-high status, consistent with the conventional biological interpretation of a majority cutoff for dominant cytotoxic T-cell infiltration. As a *post hoc* concordance check, ROC analysis using mortality as the outcome variable (AUC = 0.628) identified a data-driven Youden-optimal threshold of 60%; given the limited difference in discriminatory performance between the 50 and 60% thresholds (Youden index 0.413 vs. 0.418), this analysis supports the predefined ≥50% threshold as a reasonable and interpretable operating point rather than indicating a materially different optimum. Cases with CD8+ TIL ratio ≥50% were classified as CD8-high, and those with < 50% as CD8-low.

### 2.6 Combined immune phenotype classification

For the purpose of this classification, PD-L1 status refers specifically to PD-L1 TC positivity (≥1%, Section 2.5) — the unqualified “PD-L1 positivity” variable reported in Section 3.2.1 (62 of 137 cases, 45.3%) — and does not represent a composite or averaged score integrating the tumor cell and TIL compartments; PD-L1 TIL status is considered separately as a continuous and dichotomized variable throughout Section 3. This combined immune phenotype is a four-category cross-classification of two dichotomized variables — PD-L1 tumor cell status (positive/negative at ≥1%) and CD8+ TIL status (high/low at ≥50%). On this basis, each tumor was assigned to one of four combined PD-L1/CD8 phenotypes.

### 2.7 Statistical analysis

All statistical analyses were performed using IBM SPSS Statistics v.26.0 (IBM Corp., USA) and R software (v.4.3.1; R Foundation for Statistical Computing, Vienna, Austria). Associations between categorical variables were assessed by Pearson's chi-square test or Fisher's exact test, as appropriate. Spearman's rank correlation coefficient (ρ) was used to evaluate the relationship between PD-L1 and CD8+ TIL density as continuous variables. Independent predictors of PD-L1 positivity were identified by multivariate binary logistic regression, incorporating variables with *p* < 0.10 on univariate analysis.

Survival outcomes (overall survival [OS] and disease-free survival [DFS]) were estimated using the Kaplan-Meier method and compared between groups with the log-rank test; median OS/DFS and mortality rates were additionally compared with the Mann-Whitney U test and Fisher's exact test, respectively. For both OS and DFS, follow-up duration was used as the time variable and mortality status at last follow-up was used as the event indicator, consistent with the cross-sectional ascertainment of outcome in this retrospective cohort; a separately annotated recurrence/progression event was not available. To assess whether univariate associations between immune biomarkers (CD8-high status, PD-L1 TC positivity, and the Type I combined phenotype) and OS/DFS/mortality were confounded by clinicopathological disease burden, Cox proportional hazards regression (for OS and DFS) and binary logistic regression (for mortality) were performed, each first unadjusted and then adjusted for covariates. Candidate clinicopathological covariates (histological grade, lymphovascular invasion, nodal status, necrosis, baseline distant metastasis) were screened by univariate Cox regression against OS, and only baseline distant metastasis met the *p* < 0.10 threshold; given the small number of mortality events (*n* = 11) in this cohort, multivariable models were therefore restricted to this single covariate to maintain an adequate events-per-variable ratio, and adjusted estimates should be interpreted as exploratory given the resulting wide confidence intervals.

A two-tailed *p* < 0.05 was considered statistically significant.

## 3 Results

### 3.1 Patient and tumor characteristics

A total of 137 patients with invasive breast carcinoma were included in the final analysis. The mean age at diagnosis was 54.6 ± 13.8 years (range 13–88). The mean pathological tumor diameter was 28.6 ± 16.3 mm. The majority of tumors were invasive carcinoma of no special type (NST/ductal; 125 cases, 91.2%), with the remainder comprising ductal carcinoma with medullary features (*n* = 6), mucinous carcinoma (*n* = 3), invasive lobular carcinoma (*n* = 2), and papillary carcinoma (*n* = 1). Histological grade distribution was: Grade 1 in 28 cases (20.4%), Grade 2 in 64 cases (46.7%), and Grade 3 in 45 cases (32.8%). Lymph node metastasis was present in 78 cases (56.9%) and lymphovascular invasion (LVI) in 41 cases (29.9%). Distant metastasis was identified in 23 cases (16.8%).

By molecular subtype, 53 cases (38.7%) were Luminal A, 51 (37.2%) were TNBC, 25 (18.2%) were Luminal B, and 8 (5.8%) were HER2-enriched. For primary comparative analyses, cases were grouped as TNBC (*n* = 51, 37.2%) and hormone receptor-positive breast cancer (HR+; *n* = 86, 62.8%). By clinical stage group, 82 cases (59.9%) were classified as early stage (Stage I–II), 32 (23.4%) as locally advanced (Stage III), and 23 (16.8%) as advanced/metastatic (Stage IV). Pathological tumor stage (pT) distribution was: pT1 in 42 cases (30.7%), pT2 in 71 (51.8%), pT3 in 18 (13.1%), and pT4 in 6 (4.4%). Clinicopathological characteristics are summarized in Table 1. Interobserver agreement was substantial for PD-L1 scoring (κ = 0.78) and CD8+ TIL assessment (κ = 0.81).

**Table 1:** Clinicopathological characteristics of the study cohort (*N* = 137).

Parameter	*N* (%)
Age (years), mean ± SD (range)	54.6 ± 13.8 (13–88)
Tumor size (mm), mean ± SD	28.6 ± 16.3
Histological grade	
— Grade 1	28 (20.4%)
— Grade 2	64 (46.7%)
— Grade 3	45 (32.8%)
Histological subtype	
— Ductal NST	125 (91.2%)
— Ductal with medullary features	6 (4.4%)
— Mucinous	3 (2.2%)
— Invasive lobular	2 (1.5%)
— Papillary	1 (0.7%)
Molecular subtype	
— Luminal A	53 (38.7%)
— TNBC	51 (37.2%)
— Luminal B	25 (18.2%)
— HER2-enriched	8 (5.8%)
Binary grouping	
— HR+	86 (62.8%)
— TNBC	51 (37.2%)
Pathological tumor stage (pT)	
— pT1	42 (30.7%)
— pT2	71 (51.8%)
— pT3	18 (13.1%)
— pT4	6 (4.4%)
Clinical stage group	
— Early (Stage I–II)	82 (59.9%)
— Locally advanced (Stage III)	32 (23.4%)
— Advanced/metastatic (Stage IV)	23 (16.8%)
Lymph node status	
— Positive	78 (56.9%)
— Negative	59 (43.1%)
Lymphovascular invasion	
— Present	41 (29.9%)
— Absent	96 (70.1%)
Distant metastasis	
— Present	23 (16.8%)
— Absent	114 (83.2%)
Follow-up OS, median (range), months	43 (11–185)
Follow-up DFS, median (range), months	40 (2–185)
Mortality	11 (8.0%)

### 3.2 PD-L1 expression

#### 3.2.1 Overall prevalence

PD-L1 expression was scored and reported separately in two compartments — tumor cells (TC) and tumor-infiltrating immune cells (TIL) — as two distinct, non-interchangeable parameters, in keeping with recommendations for reproducible PD-L1 reporting. A single combined PD-L1 score integrating the two compartments into one value (for example, a Combined Positive Score) was not calculated in this study. First, PD-L1 tumor cell positivity — the unqualified “PD-L1 positivity” variable used throughout this manuscript (≥1% membranous TC staining; Section 2.5) — was identified in 62 of 137 cases (45.3%); the mean PD-L1 TC percentage (PD-L1 TC%) was 6.5% (median 0%), reflecting focal or absent tumor cell expression in the majority of cases. Second, PD-L1 TIL status was assessed and reported separately: the mean PD-L1 TIL ratio was 50.3% (median 50%), and 77 of 137 cases (56.2%) were classified as PD-L1 TIL-high (≥50%) based on the predefined majority threshold (Section 2.5). The tumor cell and TIL PD-L1 results are presented distinctly throughout the Results and Tables and should not be conflated. Where the term “combined” is used elsewhere in this manuscript (Section 2.6), it denotes the four-category immune phenotype defined by cross-classifying PD-L1 tumor cell status with CD8+ TIL status, not a combined PD-L1 score across the tumor cell and immune-cell compartments (Table 2).

**Table 2:** PD-L1 expression and association with clinicopathological parameters.

Parameter	PD-L1 negative *n* = 75	PD-L1 positive *n* = 62	*p*-value
**Molecular subtype**			0.117
— TNBC	23 (30.7%)	28 (45.2%)		
— HR+	52 (69.3%)	34 (54.8%)		
**Histological grade**			**0.020**
— Grade 1	17 (22.7%)	11 (17.7%)		
— Grade 2	41 (54.7%)	23 (37.1%)		
— Grade 3	17 (22.7%)	28 (45.2%)		
**Clinical stage group**			0.510
— Early	44 (58.7%)	38 (61.3%)		
— Locally advanced	16 (21.3%)	16 (25.8%)		
— Advanced	15 (20.0%)	8 (12.9%)		
**Lymph node status**			0.782
— Positive	44 (58.7%)	34 (54.8%)		
— Negative	31 (41.3%)	28 (45.2%)		
**Lymphovascular invasion**			0.466
— Present	20 (26.7%)	21 (33.9%)		
— Absent	55 (73.3%)	41 (66.1%)		
**Necrosis**			0.094
— Present	7 (9.3%)	13 (21.0%)		
— Absent	68 (90.7%)	49 (79.0%)		
**Distant metastasis**			0.381
— Present	15 (20.0%)	8 (12.9%)		
— Absent	60 (80.0%)	54 (87.1%)		
PDL1 TC%, median (IQR)	0.0 (0.0–0.0)	5.0 (5.0–17.5)	—
PDL1 TIL ratio%, median (IQR)	50.0 (30.0–70.0)	50.0 (40.0–70.0)	—

Chi-square test unless otherwise stated. Bold indicates p < 0.05.

#### 3.2.2 PD-L1 expression and molecular subtype

PD-L1 positivity was more frequent in TNBC than in HR+ breast cancer (54.9 vs. 39.5%), although this difference did not reach statistical significance in the binary subtype comparison (*p* = 0.117). However, PD-L1 tumor cell expression (PDL1 TC%) was significantly higher in TNBC compared with HR+ tumors (median 1.0 vs. 0.0%; Mann–Whitney *U*-test, *p* = 0.016). Among individual molecular subtypes, PD-L1 positivity rates were: TNBC 54.9% (28/51), Luminal A 39.6% (21/53), Luminal B 40.0% (10/25), and HER2-enriched 37.5% (3/8) (chi-square, *p* = 0.381), with no significant pairwise differences between any subtype pair (all Fisher's exact *p* > 0.16). PD-L1 TIL-high expression was observed in 62.7% of TNBC and 52.3% of HR+ cases (*p* = 0.379). PD-L1 TIL ratio did not differ significantly across the four molecular subtypes (Kruskal–Wallis, *p* = 0.541), with a median of 50% observed across all groups.

#### 3.2.3 PD-L1 expression and histological features

PD-L1 positivity was significantly associated with higher histological grade (chi-square, *p* = 0.020). On pairwise analysis, the difference was most pronounced between Grade 2 and Grade 3 tumors (35.9 vs. 62.2%; Fisher's exact test, *p* = 0.011). Grade 1 tumors showed intermediate positivity (39.3%), without significant differences from either Grade 2 (*p* = 0.817) or Grade 3 (*p* = 0.091). Tumors with necrosis showed numerically higher PD-L1 positivity (65.0 vs. 41.9%), with a trend approaching but not reaching significance (*p* = 0.094).

#### 3.2.4 PD-L1 expression and clinical stage

PD-L1 positivity rates across clinical stage groups were: early (Stage I–II) 46.3% (38/82), locally advanced (Stage III) 50.0% (16/32), and advanced (Stage IV) 34.8% (8/23) (chi-square, *p* = 0.510). No significant association was identified between PD-L1 positivity and pathological tumor stage (pT) (pT1 50.0%, pT2 45.1%, pT3 33.3%, pT4 50.0%; chi-square, *p* = 0.689). PD-L1 TIL ratio similarly did not differ across pT categories (Kruskal–Wallis, *p* = 0.708) or clinical stage groups (*p* = 0.079).

#### 3.2.5 PD-L1 expression and other clinicopathological parameters

No significant associations were identified between PD-L1 positivity and lymph node status (*p* = 0.782), lymphovascular invasion (*p* = 0.466), perineural invasion (*p* = 0.545), or distant metastasis (*p* = 0.381) (Table 3).

**Table 3:** CD8+ TIL density and association with clinicopathological parameters.

Parameter	CD8-Low (< 50%) *N* = 108	CD8-high (≥50%) *N* = 29	*p*-value
**Molecular subtype**			0.154
— TNBC	44 (40.7%)	7 (24.1%)		
— HR+	64 (59.3%)	22 (75.9%)		
**Histological grade**			0.301
— Grade 1	22 (20.4%)	6 (20.7%)		
— Grade 2	55 (50.9%)	9 (31.0%)		
— Grade 3	31 (28.7%)	14 (48.3%)		
**Clinical stage group**			0.621
— Early	64 (59.3%)	18 (62.1%)		
— Locally advanced	27 (25.0%)	5 (17.2%)		
— Advanced	17 (15.7%)	6 (20.7%)		
**Lymph node status**			0.996
— Positive	62 (57.4%)	16 (55.2%)		
— Negative	46 (42.6%)	13 (44.8%)		
**Lymphovascular invasion**			0.197
— Present	29 (26.9%)	12 (41.4%)		
— Absent	79 (73.1%)	17 (58.6%)		
**Distant metastasis**			0.724
— Present	17 (15.7%)	6 (20.7%)		
— Absent	91 (84.3%)	23 (79.3%)		
CD8 TIL%, median (IQR)	30.0 (20.0–30.0)	60.0 (60.0–70.0)	—
Stromal TIL%, median (IQR)	40.0 (20.0–60.0)	60.0 (40.0–70.0)	—

Chi-square test unless otherwise stated.

#### 3.2.6 Independent predictors of PD-L1 positivity

To identify independent predictors of PD-L1 positivity, variables achieving *p* < 0.10 on univariate analysis were entered into a multivariate binary logistic regression model. Histological grade (chi-square *p* = 0.020) and necrosis (Fisher's exact *p* = 0.087) met this criterion and were included. No other clinicopathological variable achieved the pre-specified *p* < 0.10 threshold on univariate screening.

On multivariate analysis (*N* = 137), histological grade as a categorical variable (Grade 1 as reference) and necrosis were entered as covariates. Grade 3 histology showed an odds ratio of 2.13 (95% CI 0.77–5.87; *p* = 0.144) compared with Grade 1, and Grade 2 showed no independent association (OR 0.82, 95% CI 0.33–2.05; *p* = 0.666). Necrosis did not independently predict PD-L1 positivity in the multivariate model (OR 1.84, 95% CI 0.64–5.33; *p* = 0.259). Although the overall model was statistically significant (likelihood ratio test χ^2^ = 9.204, df = 3; *p* = 0.027), the individual predictors did not retain independent significance after mutual adjustment, suggesting that the association between Grade 3 histology and PD-L1 positivity observed on univariate analysis reflects grade-correlated tumor characteristics rather than an independent mechanistic relationship. Results of the multivariate analysis are summarized in Table 4.

**Table 4:** Multivariate binary logistic regression: independent predictors of PD-L1 positivity, *N* = 137.

Variable	OR	95% CI	*p*-value
Histological grade (ref: Grade 1)			
— Grade 2	0.82	0.33–2.05	0.666
— Grade 3	2.13	0.77–5.87	0.144
Necrosis (present vs. absent)	1.84	0.64–5.33	0.259
Overall model	LLR χ^2^ = 9.204, df = 3	–	*p* = 0.027

OR, odds ratio; CI, confidence interval. Variables entered: histological grade (dummy-coded, Grade 1 as reference) and necrosis (binary), selected by univariate p < 0.10 criterion. McFadden R^2^ = 0.049.

### 3.3 CD8+ T lymphocyte infiltration

#### 3.3.1 Overall distribution

CD8-high infiltration (≥50%) was observed in 29 of 137 cases (21.2%). The mean CD8+ TIL ratio was 33.0% (median 30.0%, IQR 20.0–40.0%). The mean stromal TIL percentage was 44.9% (median 50.0%, IQR 20.0–60.0%).

#### 3.3.2 CD8+ TIL density and molecular subtype

CD8-high infiltration rates across individual molecular subtypes were: Luminal B 36.0% (9/25), HER2-enriched 25.0% (2/8), Luminal A 20.8% (11/53), and TNBC 13.7% (7/51) (chi-square, *p* = 0.167). On pairwise analysis, CD8-high status was significantly more frequent in Luminal B compared with TNBC (36.0 vs. 13.7%; Fisher's exact test, *p* = 0.036). No other pairwise subtype comparisons reached statistical significance (all *p* > 0.17). In the binary TNBC vs. HR+ comparison, CD8-high rates were 13.7 and 25.6%, respectively (chi-square, *p* = 0.154). Median CD8+ TIL ratios were similar between TNBC (median 30.0%, IQR 20.0%−30.0%) and HR+ tumors (median 30.0%, IQR 20.0–47.5%) (Mann–Whitney U, *p* = 0.220). CD8+ TIL ratio did not differ significantly across the four molecular subtypes (Kruskal–Wallis, *p* = 0.286), with medians of 30% (IQR 20%−40%) in Luminal A, 40% (IQR 20%−50%) in Luminal B, 30% (IQR 20%−30%) in TNBC, and 30% (IQR 20%−42%) in HER2-enriched tumors. Stromal TIL percentage showed a trend toward higher values in TNBC (median 50.0%, IQR 30.0%−70.0%) compared with HR+ tumors (median 40.0%, IQR 20.0%−60.0%), without reaching statistical significance (*p* = 0.069).

#### 3.3.3 CD8+ TIL density and lymphovascular invasion

CD8-high infiltration was numerically more frequent in tumors with LVI present compared with those without LVI (29.3% [12/41] vs. 17.7% [17/96]; chi-square, *p* = 0.197), though this difference did not reach statistical significance.

#### 3.3.4 CD8+ TIL density and clinical stage

CD8-high rates across clinical stage groups were: early 22.0% (18/82), locally advanced 15.6% (5/32), and advanced 26.1% (6/23) (chi-square, *p* = 0.621). Across pT categories, CD8-high rates were: pT1 23.8%, pT2 22.5%, pT3 11.1%, pT4 16.7% (chi-square, *p* = 0.701). Kruskal–Wallis analysis of CD8+ TIL ratios across pT categories (*p* = 0.829) and clinical stage groups (*p* = 0.309) were similarly non-significant, with a median of 30.0% across all groups.

#### 3.3.5 CD8+ TIL density and other clinicopathological parameters

No significant associations were identified between CD8-high status and histological grade (*p* = 0.301), nodal status (*p* = 0.996), necrosis (*p* = 0.875), or distant metastasis (*p* = 0.724) (Table 5).

**Table 5:** PD-L1 and CD8+ TIL associations with survival and mortality outcomes.

Parameter	OS median (mo)	DFS median (mo)	Mortality *N* (%)	p (OS)^*^	p (DFS)^*^	p (mortality)^†^
PDL1						
— Negative (*n* = 75)	46	43	6 (8.0%)	0.531	0.928	1.000
— Positive (*n* = 62)	40	38	5 (8.1%)	–	–	–
PDL1 TIL						
— Low < 50% (*n* = 60)	48	45	4 (6.7%)	0.070	0.205	0.755
— High ≥50% (*n* = 77)	39	38	7 (9.1%)	–	–	–
CD8+ TIL						
— Low < 50% (*n* = 108)	44	43	5 (4.6%)	0.256	0.354	**0.012**
— High ≥50% (*n* = 29)	39	37	6 (20.7%)	–	–	–

**Figure 2:**
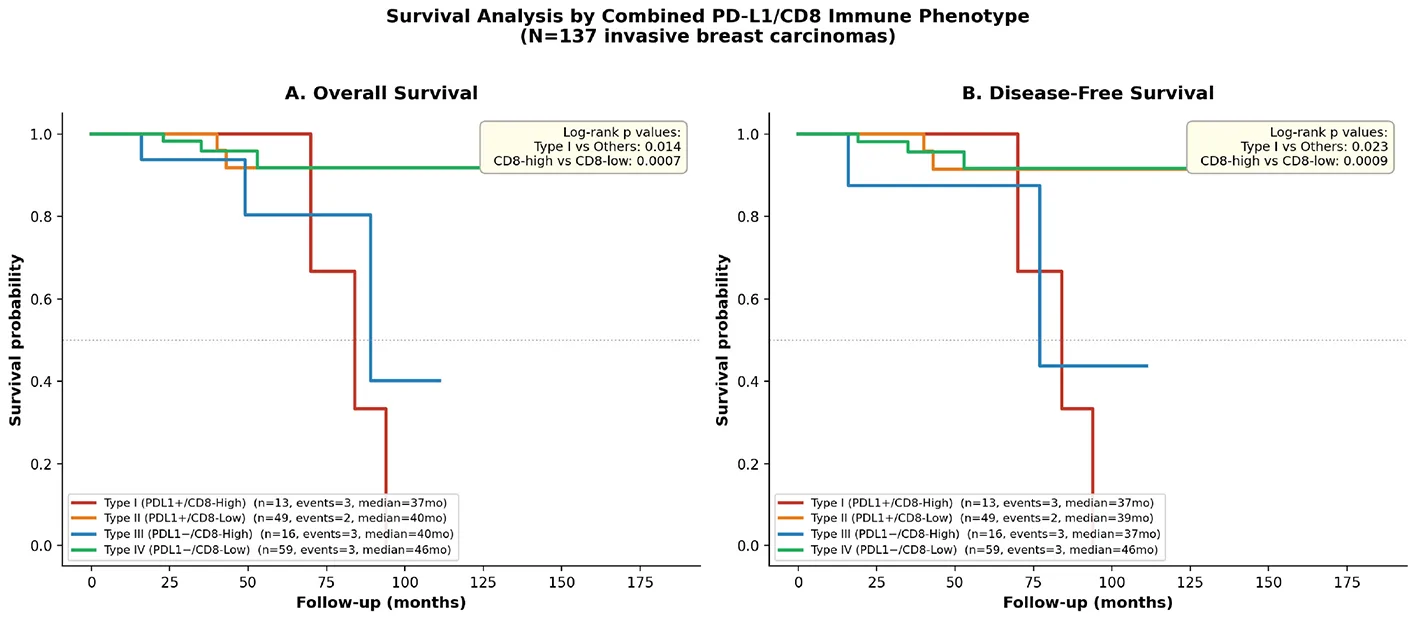
Kaplan–Meier survival curves by combined PD-L1/CD8 immune phenotype. **(A)** Overall survival and **(B)** disease-free survival stratified by Type I (PD-L1+/CD8-High), Type II (PD-L1+/CD8-Low), Type III (PD-L1–/CD8-High), and Type IV (PD-L1–/CD8-Low) phenotype. Log-rank *p*-values shown for Type I vs. others and Type I vs. Type IV comparisons.

Mann–Whitney U–test. ^*^Denotes the Mann–Whitney U test used for comparisons of overall survival (OS) and disease-free survival (DFS). ^†^Fisher's exact test used for mortality comparisons. Bold indicates p < 0.05. OS and DFS represent follow-up duration used as the time variable; mortality is a cross-sectional endpoint. Kaplan–Meier analysis by combined immune phenotype is presented in Table 6 and Figure 2.

Unadjusted and adjusted Cox proportional hazards and logistic regression models for these same associations are presented in Table 7.

#### 3.3.6 PD-L1 and CD8+ TIL density-based survival outcomes

Overall survival (OS) and disease-free survival (DFS) did not differ significantly between CD8-high and CD8-low groups (median OS 39 vs. 44 months, Mann–Whitney *p* = 0.256; median DFS 37 vs. 43 months, *p* = 0.354). However, mortality was significantly higher in the CD8-high group: 6 of 29 CD8-high cases (20.7%) died during follow-up, compared with 5 of 108 CD8-low cases (4.6%) (Fisher's exact test, *p* = 0.012). PD-L1 positivity was not significantly associated with OS (median 40 vs. 46 months; *p* = 0.531), DFS (*p* = 0.928), or mortality (PDL1+ 8.1 vs. PDL1– 8.0%; *p* = 1.000). PD-L1 TIL-high status showed a borderline trend toward shorter OS (median 39 vs. 48 months; *p* = 0.070) that did not reach statistical significance. On continuous correlation analysis, neither CD8+ TIL ratio nor PD-L1 TIL ratio showed significant Spearman correlations with OS (ρ = −0.141, *p* = 0.101 and ρ = −0.082, *p* = 0.342, respectively) or DFS (ρ = −0.124, *p* = 0.149 and ρ = −0.030, *p* = 0.724, respectively). These survival data, together with the Kaplan–Meier analyses presented in Section 3.5.1 (Table 6), employ follow-up duration as the time variable and cross-sectional mortality as the event outcome rather than fully annotated time-to-event data; corresponding unadjusted and adjusted Cox proportional hazards and logistic regression models are presented in Section 3.5.2 (Table 7), subject to the same data-structure caveat. The observed associations therefore represent exploratory endpoints that gain mechanistic coherence when interpreted in the context of the combined immune phenotype analysis — specifically, the exhaustion-driven immune dysfunction model elaborated in Section 4.5 — rather than as independent prognostic parameters.

**Table 6:** Survival outcomes by combined PD-L1/CD8 immune phenotype (*N* = 137).

Phenotype	*N* (%)	Mortality *N* (%)	Median OS, mo (IQR)	Median DFS, mo (IQR)	Log-rank vs. Type IV (OS)	Log-rank vs. Type IV (DFS)
**Type I (PD-L1+/CD8-High)**	13 (9.5%)	**3 (23.1%)**	37 (36–61)	37 (36–61)	***p*** **=** **0.014**	***p*** **=** **0.021**
Type II (PD-L1+/CD8-Low)	49 (35.8%)	2 (4.1%)	40 (29–76)	39 (29–71)	*p* = 0.925	*p* = 0.893
Type III (PD-L1–/CD8-High)	16 (11.7%)	3 (18.8%)	40 (32–55)	37 (22–55)	*p* = 0.050	*p* = 0.049
Type IV (PD-L1–/CD8-Low)	59 (43.1%)	3 (5.1%)	46 (34–82)	46 (31–76)	Reference	Reference

OS, overall survival; DFS, disease-free survival; IQR, interquartile range. Log-rank p-values are for pairwise comparison with Type IV (reference). Overall chi-square for phenotype vs. mortality: p = 0.042. Type I vs. all others combined: log-rank OS p = 0.014, DFS p = 0.023. OS and DFS represent follow-up duration; mortality is a cross-sectional endpoint. Corresponding unadjusted and adjusted Cox proportional hazards and logistic regression models are presented in Table 7; given the small event count (n = 11), all estimates in both tables should be interpreted as exploratory.

**Table 7:** Unadjusted and adjusted cox proportional hazards and logistic regression models for OS, DFS, and mortality.

Outcome/predictor	Unadjusted HR/OR (95% CI)	Unadjusted *p*	Adjusted HR/OR (95% CI)*	Adjusted *p*	Model
OS — CD8-high	6.29 (1.88–21.01)	0.003	5.06 (1.47–17.43)	0.010	Cox
OS — PD-L1 TC+	1.05 (0.32–3.45)	0.933	1.24 (0.38–4.08)	0.725	Cox
OS — Type I phenotype	4.61 (1.21–17.63)	0.025	3.68 (0.92–14.73)	0.066	Cox
DFS — CD8-high	6.06 (1.82–20.14)	0.003	4.25 (1.22–14.85)	0.023	Cox
DFS — Type I phenotype	4.14 (1.09–15.73)	0.037	2.66 (0.67–10.57)	0.165	Cox
Mortality — CD8-high	5.37 (1.51–19.13)	0.010	6.25 (1.48–26.39)	0.013	Logistic
Mortality — Type I phenotype	4.35 (0.99–19.03)	0.051	4.62 (0.85–25.08)	0.076	Logistic

HR, hazard ratio (Cox proportional hazards regression, OS/DFS); OR, odds ratio (binary logistic regression, mortality); CI, confidence interval. ^*^Adjusted for baseline distant metastasis, the sole covariate meeting the pre-specified p < 0.10 univariate screening criterion (Section 2.7); adjustment was restricted to one covariate given the small number of mortality events (n = 11). Estimates should be interpreted as exploratory given the resulting wide confidence intervals.

### 3.4 Correlation between PD-L1 expression and CD8+ TIL density

A significant positive correlation was identified between PD-L1 TIL ratio and CD8+ TIL ratio (Spearman ρ = 0.453, *p* < 0.001), indicating that tumors with higher CD8+ T cell infiltration exhibited proportionally greater PD-L1 expression within the immune cell compartment. In contrast, PD-L1 tumor cell percentage (PDL1 TC%) showed no significant correlation with CD8+ TIL ratio (ρ = −0.021, *p* = 0.812), suggesting that tumor cell-intrinsic PD-L1 expression operates independently of the cytotoxic immune infiltrate. Stromal TIL percentage was also positively correlated with CD8+ TIL ratio (ρ = 0.201, *p* = 0.019). These findings are consistent with an adaptive immune resistance mechanism, whereby CD8+ T cell infiltration drives PD-L1 upregulation in tumor-associated immune cells rather than in tumor cells themselves. When PD-L1 expression was evaluated as a binary variable, no significant correlation with CD8+ TIL ratio was identified (ρ = 0.012, *p* = 0.890), suggesting that the threshold-based positivity classification does not capture the continuous relationship between immune cell PD-L1 expression and CD8+ infiltration.

### 3.5 Combined PD-L1/CD8 immune phenotype classification

Based on combined PD-L1 and CD8+ TIL (≥50%) status, cases were assigned to four immune phenotypes. The most frequent phenotype was Type IV (PD-L1–/CD8-Low; 59 cases, 43.1%), representing the immunologically “cold” tumor phenotype, followed by Type II (PD-L1+/CD8-Low; 49 cases, 35.8%), Type III (PD-L1–/CD8-High; 16 cases, 11.7%), and Type I (PD-L1+/CD8-High; 13 cases, 9.5%). The adaptive immune resistance phenotype (Type I) was observed in 9.5% of all cases.

The distribution of combined immune phenotypes did not differ significantly between TNBC and HR+ subgroups (chi-square, *p* = 0.125). The adaptive resistance phenotype (Type I) was observed in 7.8% of TNBC cases (4/51) vs. 10.5% of HR+ cases (9/86), while the immunologically cold phenotype (Type IV) was present in 39.2% of TNBC (20/51) and 45.3% of HR+ tumors (39/86). Phenotype distribution did not vary significantly by clinical stage group (chi-square, *p* = 0.776) or by pathological tumor stage (*p* = 0.689). The combined phenotype classification and its distribution by subtype and stage are presented in Table 8.

**Table 8:** Combined PD-L1/CD8 immune phenotype distribution by molecular subtype and clinical stage.

Phenotype	Total *N* = 137	TNBC *N* = 51	HR+ *N* = 86	Early *N* = 82	Loc. adv. *N* = 32	Advanced *N* = 23
Type I: PD-L1+/CD8-High	13 (9.5%)	4 (7.8%)	9 (10.5%)	8 (9.8%)	2 (6.3%)	3 (13.0%)
Type II: PD-L1+/CD8-Low	49 (35.8%)	24 (47.1%)	25 (29.1%)	30 (36.6%)	14 (43.8%)	5 (21.7%)
Type III: PD-L1–/CD8-High	16 (11.7%)	3 (5.9%)	13 (15.1%)	10 (12.2%)	3 (9.4%)	3 (13.0%)
Type IV: PD-L1–/CD8-Low	59 (43.1%)	20 (39.2%)	39 (45.3%)	34 (41.5%)	13 (40.6%)	12 (52.2%)
*p*-value (subtype)	—	0.125	–	–	–	–
*p*-value (stage)	—	–	–	0.776	–	–

Subtype comparison: chi-square across four phenotypes × TNBC vs. HR+. Stage comparison: chi-square across four phenotypes × three stage groups.

#### 3.5.1 Survival outcomes by combined immune phenotype

Survival outcomes were compared across the four immune phenotype groups. The adaptive immune resistance phenotype (Type I: PD-L1+/CD8-High) was associated with the shortest overall survival (OS) and highest mortality among all phenotypes. Mortality was observed in 3 of 13 Type I cases (23.1%), compared with 2 of 49 Type II cases (4.1%), 3 of 16 Type III cases (18.8%), and 3 of 59 Type IV cases (5.1%) (chi-square, *p* = 0.042). Median OS was shortest in Type I (37 months; IQR 36–61) and Type III (40 months; IQR 32–55) tumors, compared with Type II (40 months; IQR 29–76) and Type IV (46 months; IQR 34–82). On Kaplan–Meier analysis using follow-up duration as the time variable and cross-sectional mortality as the event (Section 2.7), the Type I phenotype showed a lower follow-up-based survival estimate than all other phenotypes combined (log-rank *p* = 0.014) and specifically compared with Type II (log-rank *p* = 0.006) and Type IV (log-rank *p* = 0.014); given that only 11 deaths occurred across the entire cohort (3 within the Type I subgroup), these log-rank comparisons should be interpreted as exploratory associations rather than definitive survival differences. Similarly, Type I showed the shortest disease-free survival (DFS; median 37 months), with nominally significant log-rank differences from Type II (*p* = 0.007), Type IV (*p* = 0.021), and all other phenotypes combined (*p* = 0.023), subject to the same interpretive caution. The Type III phenotype (PD-L1–/CD8-High) also showed elevated mortality (18.8%) and a borderline lower OS estimate compared with Type IV (log-rank *p* = 0.050), consistent with CD8-driven immune activity in the absence of PD-L1–mediated adaptive resistance. The survival outcomes of combined immune phenotypes are summarized in Table 5 and illustrated in Figure 2.

These findings extend the prognostic relevance of the combined immune phenotype classification beyond its descriptive utility. The convergence of high PD-L1 expression and high CD8+ TIL density in Type I tumors, rather than conferring immune-mediated tumor control, was paradoxically associated with the worst survival outcome in this cohort. This is consistent with the adaptive immune resistance model, in which PD-L1 upregulation suppresses the cytotoxic function of infiltrating CD8+ T cells, rendering the immune response ineffective despite its quantitative abundance.

Given that this observation rests on only 3 deaths within the Type I subgroup (*n* = 13), and is based on follow-up duration and cross-sectional mortality rather than formally annotated time-to-event data (Section 3.5.2), it should be regarded as an exploratory, hypothesis-generating association rather than evidence sufficient to inform clinical prioritization of Type I tumors for checkpoint blockade; such a recommendation would require confirmation in a substantially larger, prospectively followed cohort with adequately powered multivariable survival modeling.

#### 3.5.2 Adjusted analysis for confounding by disease burden

Because the univariate associations reported above could be confounded by underlying disease burden, Cox proportional hazards regression (OS, DFS) and binary logistic regression (mortality) were performed for the three key immune biomarkers — CD8-high status, PD-L1 TC positivity, and the Type I (PD-L1+/CD8-high) combined phenotype — both unadjusted and adjusted for baseline distant metastasis, the only clinicopathological covariate meeting the pre-specified *p* < 0.10 univariate screening criterion (Section 2.7). Results are summarized in Table 6.

CD8-high status remained significantly associated with inferior OS (adjusted HR 5.06, 95% CI 1.47–17.43; *p* = 0.010), inferior DFS (adjusted HR 4.25, 95% CI 1.22–14.85; *p* = 0.023), and higher mortality (adjusted OR 6.25, 95% CI 1.48–26.39; *p* = 0.013) after adjustment for baseline distant metastasis, indicating that this association is not fully explained by more advanced disease at presentation. PD-L1 TC positivity was not significantly associated with OS in either the unadjusted (HR 1.05, *p* = 0.933) or adjusted model (HR 1.24, *p* = 0.725), consistent with the univariate findings in Section 3.3.6. In contrast, the Type I phenotype, which was significantly associated with inferior OS (unadjusted HR 4.61, 95% CI 1.21–17.63; *p* = 0.025) and DFS (unadjusted HR 4.14, 95% CI 1.09–15.73; *p* = 0.037) on univariate Cox regression, was attenuated to borderline or non-significance after adjustment for baseline distant metastasis (OS: adjusted HR 3.68, 95% CI 0.92–14.73, *p* = 0.066; DFS: adjusted HR 2.66, 95% CI 0.67–10.57, *p* = 0.165; mortality: adjusted OR 4.62, 95% CI 0.85–25.08, *p* = 0.076). This attenuation indicates that a meaningful component of the apparent Type I survival disadvantage is attributable to a higher baseline burden of metastatic disease among Type I cases rather than to the combined immune phenotype independently, and the exhaustion-related interpretation of this phenotype (Section 4.5) is revised accordingly to reflect this reduced statistical certainty. Given the small number of mortality events (n = 11) underlying all models, the wide confidence intervals reflect limited statistical power, and these adjusted estimates should be regarded as exploratory pending confirmation in a larger, prospectively assembled cohort.

### 3.6 Stromal TIL density and its relationship with PD-L1 and CD8+ expression

Stromal TIL density was evaluated in all 137 cases (mean 44.9%, median 50.0%, IQR 20.0%−60.0%). Cases were categorized by the modified IWG criteria: low (0%−10%): 17 cases (12.4%); moderate (10%−40%): 48 cases (35.0%); high (>40%): 72 cases (52.6%). For binary analyses, cases were dichotomized as stromal TIL-high (>40%, *n* = 72) and stromal TIL-low ( ≤ 40%, *n* = 65).

CD8+ TIL ratio was significantly higher in the stromal TIL-high group compared with the stromal TIL-low group (median 30 vs. 30%; Mann–Whitney *p* = 0.027), and a trend toward greater CD8-high categorical status was observed (27.8 vs. 13.8%; Fisher's exact test, *p* = 0.060). In contrast, PD-L1 TIL ratio (median 50% in both groups; *p* = 0.948) and PD-L1 positivity (50.0 vs. 40.0%; *p* = 0.303) did not differ significantly between stromal TIL groups, indicating that overall stromal immune density is coupled to cytotoxic T cell infiltration but not to PD-L1 expression. On continuous analysis, these findings were corroborated by the significant Spearman correlation between stromal TIL density and CD8+ TIL ratio (ρ = 0.201, *p* = 0.019), in the absence of significant correlations with PD-L1 TIL ratio (ρ = 0.103, *p* = 0.232) or PD-L1 TC% (ρ = 0.119, *p* = 0.166). Stromal TIL density did not differ significantly across clinical stage groups (Kruskal–Wallis, *p* = 0.889). IWG-based grouping results are summarized in Table 9.

**Table 9:** Stromal TIL density by IWG category and associations with PD-L1 and CD8+ TIL expression.

Parameter	Low TIL (0–10%) *N* = 17	Moderate TIL (10%−40%) *N* = 48	High TIL (>40%) *N* = 72	*p*-value
Stromal TIL%, median (IQR)	10 (10–10)	30 (20–32)	60 (50–70)	—
PD-L1 TIL ratio%, median (IQR)	50 (30–70)	50 (30–70)	50 (30–70)	0.980
CD8 TIL%, median (IQR)	30 (20–40)	28 (20–40)	30 (20–50)	0.070
PD-L1 +, N (%)	7 (41.2%)	19 (39.6%)	36 (50.0%)	0.499

p-values: Kruskal–Wallis for continuous variables; chi-square for categorical. Spearman correlations with stromal TIL as continuous variable: CD8 TIL ratio ρ = 0.201 (p = 0.019); PD-L1 TIL ratio ρ = 0.103 (p = 0.232); PD-L1 TC% ρ = 0.119 (p = 0.166).

## 4 Discussion

The present multicenter retrospective study characterized PD-L1 expression and CD8+ TIL infiltration across 137 invasive breast carcinomas spanning the full spectrum of molecular subtypes and clinical stages. Four principal findings emerged. First, PD-L1 positivity was significantly associated with higher histological grade but not with pathological tumor stage (pT), clinical stage group, or lymph node status, indicating that PD-L1 upregulation is coupled to intrinsic tumor biology rather than anatomical disease extent. Second, a significant positive correlation between PD-L1 TIL ratio and CD8+ TIL density (Spearman ρ = 0.453, *p* < 0.001) was identified in the absence of any correlation between PD-L1 tumor cell expression and CD8+ density, consistent with an adaptive immune resistance mechanism. Third, CD8-high status (≥50%) was associated with higher mortality (20.7 vs. 4.6%; *p* = 0.012) and was more frequent in Luminal B than in TNBC tumors (36.0 vs. 13.7%; *p* = 0.036); both findings are contrary to prevailing assumptions; while direct exhaustion markers were not assessed, subtype-specific T cell exhaustion is, among plausible explanations, the most coherent hypothesis, as discussed in Section 4.5. Fourth, the predominance of the immunologically cold phenotype (Type IV: PD-L1–/CD8-Low, 43.1%) across this mixed-subtype cohort highlights the limited immunological activity of most archival breast carcinomas, with direct implications for patient selection in checkpoint blockade strategies (Table 10).

**Table 10:** Classification and biological interpretation of the combined PD-L1/CD8 immune phenotypes.

Phenotype	PD-L1	CD8	Biological Interpretation
Type I	+	High	Adaptive immune resistance
Type II	+	Low	Constitutive/oncogenic PD-L1 upregulation
Type III	-	High	Active cytotoxicity without PD-L1 evasion
Type IV	-	Low	Immunologically “cold” tumor

### 4.1 PD-L1 expression: prevalence and clinicopathological associations

The overall PD-L1 positivity rate of 45.3% in the present cohort is consistent with the broad range reported in the literature, which spans 20%−65% depending on the scoring system, antibody clone, and patient population (18, 19). The higher positivity rate in TNBC (54.9%) compared with HR+ tumors (39.5%), though statistically non-significant in the binary comparison (*p* = 0.117), is directionally concordant with established immunobiological differences between subtypes. Critically, PD-L1 tumor cell percentage (TC%) was significantly higher in TNBC at the continuous level (median 1.0 vs. 0.0%; *p* = 0.016), reinforcing the known immunogenic character of TNBC and the limited sensitivity of binary cutoffs in mixed-subtype cohorts. Among individual molecular subtypes, PD-L1 positivity rates did not differ significantly (*p* = 0.381), and no pairwise subtype comparison reached significance, suggesting that PD-L1 expression has limited subtype discriminating capacity. PD-L1 TIL ratios were uniformly distributed across all subtypes (Kruskal–Wallis *p* = 0.541), with a median of 50% regardless of molecular group, underscoring the compartment-specific nature of immune regulation.

The significant association between PD-L1 positivity and Grade 3 histology (*p* = 0.020; Grade 2 vs. 3 pairwise *p* = 0.011) supports the hypothesis that increased mutational burden and neoantigenic load in poorly differentiated tumors promotes immune activation and secondary PD-L1 induction (12). The trend toward higher PD-L1 in tumors with necrosis (65.0 vs. 41.9%; *p* = 0.094) is biologically plausible, as hypoxic and necrosis-associated danger signals within the tumor microenvironment promote transcriptional upregulation of PD-L1.

In contrast, PD-L1 positivity did not vary significantly across pathological tumor stage (pT1–pT4; *p* = 0.689) or clinical stage groups (*p* = 0.510), and was independent of lymph node status, LVI, and distant metastasis. This stage-independence is consistent with prior series (11, 12) and indicates that PD-L1 expression is determined primarily by the immunological state of the primary tumor microenvironment rather than by the extent of anatomical spread.

On multivariate logistic regression incorporating histological grade and necrosis the two variables achieving *p* < 0.10 on univariate screening neither retained independent significance after mutual adjustment (Grade 3 vs. Grade 1: OR 2.13, 95% CI 0.77–5.87, *p* = 0.144; necrosis: OR 1.84, 95% CI 0.64–5.33, *p* = 0.259), despite overall model significance (LLR *p* = 0.027). This suggests that grade-associated PD-L1 upregulation operates through correlated tumor microenvironmental features rather than a single dominant independent pathway, and that larger cohorts with adequate power across grade strata are required for definitive multivariate characterization.

### 4.2 CD8+ TIL density: distribution and subtype associations

CD8-high infiltration (≥50%) was identified in 21.2% of cases. The application of a predefined ≥50% majority threshold, confirmed by *post hoc* ROC concordance analysis using mortality as the outcome variable (AUC = 0.628), yielded a biologically interpretable definition of CD8-dominant immune infiltration.

A noteworthy finding was the significantly higher CD8-high rate in Luminal B compared with TNBC tumors (36.0 vs. 13.7%; Fisher's exact *p* = 0.036). This is contrary to the broadly held assumption that TNBC harbors the most abundant CD8+ TIL infiltration — an assumption largely derived from studies applying lower absolute cell count thresholds (4, 5). At the ≥50% ratio threshold, representing cytotoxic T cell predominance, Luminal B tumors exhibited the highest rate among all subtypes, while TNBC showed the lowest. This observation may partially reflect the immunomodulatory effects of progesterone receptor loss and high Ki-67 in Luminal B disease, and warrants specific investigation in the context of CDK4/6 inhibitor-mediated T cell priming, which has been demonstrated to increase CD8+ intratumoral density and reduce regulatory T cell proliferation in preclinical models (20). The small number of CD8-high cases (*n* = 29 total; Luminal B *n* = 9, TNBC *n* = 7, HER2-enriched *n* = 2) limits the statistical power of pairwise subtype comparisons; the observed Luminal B vs. TNBC difference (*p* = 0.036) should therefore be regarded as a hypothesis-generating signal rather than a confirmatory finding, and replication in a larger, prospectively collected cohort is required before clinical conclusions can be drawn.

CD8+ TIL density was independent of pathological tumor stage (pT; *p* = 0.701), clinical stage group (*p* = 0.621), histological grade, nodal status, and distant metastasis — consistent with the hypothesis that cytotoxic immune infiltration represents a microenvironmental property determined by tumor immunogenicity rather than disease burden.

The discordance between the categorical CD8-high/low classification and the underlying continuous CD8+ TIL ratio distribution warrants explicit comment. Although CD8-high status was significantly more frequent in Luminal B than TNBC tumors when dichotomized at the ≥50% threshold (Fisher's exact *p* = 0.036), the continuous CD8+ TIL ratio itself did not differ significantly across the four molecular subtypes (Kruskal–Wallis *p* = 0.286) or between TNBC and HR+ tumors as a binary group (Mann–Whitney *p* = 0.220), and stromal TIL percentage — a lineage-independent, H&E-based immune density measure — showed, if anything, the opposite directional trend, trending higher in TNBC (*p* = 0.069). This pattern indicates that the categorical association is driven by where a relatively small number of high-ratio cases (*n* = 29 CD8-high overall) happen to fall relative to a single cohort-derived cutoff, rather than by a systematic shift in the overall CD8+ infiltration distribution between subtypes. Because this ≥50% cutoff, although predefined *a priori* (Section 2.5), operates as a single fixed cut-point applied to a modestly sized subgroup (Luminal B CD8-high *n* = 9), such divergence between the dichotomized and continuous representations of the same variable is an expected consequence of the sensitivity of any fixed threshold to small-sample cutoff placement, rather than necessarily reflecting a genuine subtype-specific biological ceiling effect on CD8+ infiltration. This does not invalidate the observation, but it substantially tempers confidence that Luminal B tumors possess a truly distinct high-density CD8+ phenotype relative to TNBC, as opposed to the association reflecting cutoff placement within a small subgroup (Luminal B CD8-high *n* = 9). Independent validation of both the threshold and the subtype association in an external cohort is required before concluding that this represents a genuine biological difference rather than a statistical artifact of dichotomization.

### 4.3 CD8+ TIL density and mortality

CD8-high status (≥50%) was associated with higher mortality (20.7 vs. 4.6%; Fisher's exact *p* = 0.012), a finding that appears paradoxical relative to the established favorable prognostic role of CD8+ TIL infiltration in breast cancer (4, 5). Importantly, this association was not attributable to more advanced disease at presentation: CD8-high status remained significantly associated with inferior OS, inferior DFS, and higher mortality after adjustment for baseline distant metastasis (Section 3.5.2, Table 6), the only clinicopathological covariate meeting the pre-specified confounder screening criterion. This adjustment-robust persistence strengthens confidence that the association reflects a genuine biological phenomenon rather than confounding by disease burden, although the wide confidence intervals arising from the small number of mortality events (*n* = 11) warrant caution in interpreting the precise magnitude of effect. The mechanistic basis for this observation — encompassing T cell exhaustion, subtype-specific immune dynamics, and methodological considerations — is addressed in the context of the combined immune phenotype analysis in Section 4.5.

### 4.4 PD-L1/CD8 correlation and adaptive immune resistance

The central immunobiological finding of this study is the significant positive correlation between PD-L1 TIL ratio and CD8+ TIL density (Spearman ρ = 0.453, *p* < 0.001). This correlation pattern high CD8+ infiltration co-occurring with high PD-L1 expression in immune cells, but not in tumor cells (ρ = −0.021, *p* = 0.812) is the immunohistochemical signature of adaptive immune resistance, as conceptualized by Taube et al. (9). Under this framework, IFN-γ secreted by cytotoxic CD8+ T cells engages JAK/STAT1 signaling in adjacent immune and tumor cells, transcriptionally upregulating PD-L1 as a counter-regulatory mechanism. The uncoupling of PD-L1 tumor cell expression from CD8+ infiltration indicates that constitutive, oncogene-driven PD-L1 expression on tumor cells mediated by PI3K/AKT, MYC amplification, or PTEN loss represents a biologically distinct mechanism not reflected in the immune cell compartment (10).

This dissociation has critical implications for biomarker strategy. PD-L1 scoring aggregates tumor cell and immune cell staining and may thereby obscure mechanistically distinct regulatory pathways. The present data suggest that PD-L1 expression specifically in tumor-infiltrating immune cells, evaluated in conjunction with CD8+ density, more accurately captures the adaptive resistance state. This interpretation is supported by Wang et al., who demonstrated that combined PD-L1+/CD8-high status was the sole independent predictor of recurrence-free survival in basal-like breast cancer (HR 0.12, *p* = 0.010) (11), and by Hou et al., who identified the synergistic prognostic value of co-expressed PD-L1 and CD8+ in HER2-positive disease (12).

### 4.5 Combined immune phenotype classification

The four-phenotype classification confirmed that combined PD-L1/CD8 immune phenotype is a microenvironmental property independent of both molecular subtype and clinical stage — a finding with direct implications for patient selection strategies, as discussed below.

The predominance of Type IV and Type II phenotypes (collectively 78.9%) indicates that the majority of tumors in this mixed-subtype cohort lack the active cytotoxic infiltration that underpins checkpoint inhibitor efficacy. The adaptive resistance phenotype (Type I) — the subset that, by IHC criteria alone, would conventionally be considered most biologically positioned for checkpoint blockade response — comprised only 9.5% of cases overall, without subtype enrichment, a finding that supports the need for direct immune phenotyping rather than subtype-based patient selection; as detailed below, however, this phenotype paradoxically showed the least favorable unadjusted survival, underscoring that IHC-based positioning does not equate to a validated indication for treatment prioritization. The relatively higher Type IV rate in HR+ tumors (45.3 vs. 39.2% in TNBC), though not statistically significant, is consistent with the known immunological quiescence of HR+ disease and the limited benefit of single-agent checkpoint blockade in this subtype in available clinical data (17).

A notable finding of the present study is that the Type I (PD-L1+/CD8-High) phenotype, rather than conferring the best prognosis as might be expected from the adaptive immune resistance framework, was associated with the highest mortality (23.1%) and shortest median OS (37 months) across all four phenotype groups on unadjusted comparison (chi-square *p* = 0.042; log-rank Type I vs. all others *p* = 0.014). However, as detailed in Section 3.5.2, this univariate association was attenuated to borderline or non-significance after adjustment for baseline distant metastasis (adjusted HR 3.68, 95% CI 0.92–14.73, *p* = 0.066 for OS; adjusted HR 2.66, 95% CI 0.67–10.57, *p* = 0.165 for DFS), indicating that a meaningful portion of the apparent Type I survival disadvantage reflects a higher burden of metastatic disease at presentation among Type I cases rather than a fully independent effect of the combined immune phenotype. The exhaustion-driven mechanistic interpretation developed below should therefore be read as a plausible, hypothesis-generating explanation for the unadjusted signal rather than an adjustment-robust, established finding, pending confirmation in a larger cohort with greater statistical power for multivariable modeling. This unadjusted paradox is nonetheless consistent with and mechanistically explicable by the emerging concept of CD8+ T cell exhaustion in the breast cancer tumor microenvironment. Exhausted CD8+ T cells (Tt_ex_) are characterized by progressive loss of effector function, sustained co-expression of inhibitory receptors including PD-1 and CD39, reduced cytokine production capacity, and transcriptionally distinct epigenetic states that resist reversal (14, 15). Critically, tumors harboring abundant Tt_ex_ display a paradoxical immune microenvironment: high CD8+ T cell infiltration and elevated IFN-γ signaling co-occur with high PD-L1 expression, creating the immunohistochemical signature of adaptive immune resistance while producing quantitatively poor antitumor immunity (14).

This interpretation is directly supported by Egelston et al., who demonstrated in a prospective cohort of breast cancer patients that tumors with abundant CD8+ Tt_ex_ exhibit a tumor microenvironment marked by amplified IFN-γ signaling and higher PD-L1 expression, and that — paradoxically — high CD8+ Tt_ex_ infiltration was associated with decreased overall survival in ER+ breast cancer patients, but not in TNBC (14). This subtype-specific divergence is directly relevant to the present cohort, in which HR+ tumors comprise 62.8% of cases: the adverse prognostic signal of the Type I phenotype observed here likely reflects the exhaustion-driven immunosuppression that is characteristic of ER+ disease rather than the immunologically active adaptive resistance state that confers benefit in TNBC (14, 15). Xie et al. further established in a comprehensive 2024 review that CD8+ T cell exhaustion in the breast cancer tumor microenvironment operates through multiple convergent mechanisms — inhibitory receptor upregulation, immunosuppressive cytokine signaling, metabolic reprogramming, and epigenetic silencing of effector programs — and that Tt_ex_ production is positively correlated with poor prognosis and reduced response rates to immunotherapy (15). The TWIST1/PD-L1 axis provides a further molecular link: TWIST1-mediated transcriptional upregulation of PD-L1 in breast cancer cells drives CD8+ T cell exhaustion through sustained PD-1/PD-L1 engagement, and blockade of this axis reinvigorates CD8+ T cell cytotoxic function (21).

Taken together, these data reframe the prognostic interpretation of the Type I phenotype in mixed-subtype breast cancer cohorts. Rather than uniformly representing immune-active tumors primed for checkpoint blockade response, Type I tumors in a predominantly HR+ cohort may instead represent a state of exhaustion-driven immune paralysis in which quantitative CD8+ infiltration and PD-L1 co-expression reflect chronic antigen stimulation and T cell dysfunction rather than active cytotoxic surveillance. This interpretation does not contradict the favorable prognostic role of combined PD-L1+/CD8-high status reported in TNBC and basal-like disease (11) — a finding in which TNBC-specific immune activation rather than exhaustion is likely the dominant mechanism — but rather emphasizes that the prognostic and predictive meaning of the combined immune phenotype is subtype-dependent and cannot be generalized across molecularly heterogeneous cohorts. Prospective studies incorporating CD8+ T cell exhaustion markers (PD-1, CD39, TOX, LAG-3, TIM-3) alongside PD-L1 and CD8 IHC are required to dissect these overlapping phenotypic states and optimize checkpoint blockade patient selection.

### 4.6 Stromal TIL density

Stromal TIL density was positively correlated with CD8+ TIL ratio (ρ = 0.201, *p* = 0.019), confirming that H&E-based immune density assessment is a meaningful surrogate for cytotoxic T cell presence. However, stromal TIL was not significantly associated with PD-L1 expression (TIL ratio *p* = 0.948; *p* = 0.303), indicating that overall immune cellularity does not predict PD-L1 status. Importantly, CD8+ TIL ratio was significantly higher in the stromal TIL-high group on continuous analysis (Mann–Whitney *p* = 0.027), consistent with a model in which quantitative immune density by H&E can serve as an accessible screening indicator for cytotoxic infiltration in settings where CD8 IHC is not available. The predominance of high-TIL cases (52.6%) in this cohort likely reflects the TNBC and HER2-enriched representation, subtypes known to harbor the greatest stromal immune infiltrates.

### 4.7 Limitations

Several limitations warrant consideration. First, the retrospective archival design spanning 16 years (2009–2025) introduces potential variability in tissue fixation and antigen preservation, notwithstanding standardized automated protocols across both centers. Second, PD-L1 expression was assessed from a single representative block, which does not capture intratumoral spatiotemporal heterogeneity.

Third, the Kaplan–Meier, log-rank, and Cox/logistic regression analyses presented in this study (Sections 3.3.6, 3.5.1, 3.5.2) all use follow-up duration as the time variable and cross-sectional mortality status as the event indicator, rather than formally annotated time-to-event data with independently verified censoring; this approach generates directional, exploratory prognostic signals that are mechanistically interpretable but not interchangeable with hazard-based outcomes derived from prospectively annotated event-time data. Prospective replication with fully annotated event-time data is the necessary next step.

Fourth, although the PD-L1 TC (≥1%), PD-L1 TIL (≥50%), and CD8+ TIL (≥50%) thresholds were predefined *a priori* on biological and clinical grounds rather than optimized within this cohort, and ROC analysis confirmed close concordance with these predefined values, formal external validation in an independent cohort remains necessary before any of these thresholds can be proposed as definitive reproducible clinical criteria. Fifth, the limited number of CD8-high cases overall (*n* = 29), and within individual subtypes (Luminal B *n* = 9, TNBC *n* = 7), constrained statistical power for subgroup comparisons; the Luminal B vs. TNBC difference (*p* = 0.036) is therefore best regarded as a biologically plausible hypothesis requiring confirmation in a larger, prospectively assembled series.

Sixth, the exhaustion-driven interpretation of the Type I phenotype's adverse prognostic association is inferential in nature, as direct exhaustion markers (PD-1, CD39, TOX, LAG-3, TIM-3) were not assessed. The IHC pattern of PD-L1+/CD8-high co-expression with poor survival is consistent with but does not confirm T cell exhaustion; alternative mechanisms including regulatory T cell infiltration or myeloid-mediated immunosuppression cannot be excluded on the basis of the present data.

Seventh, the adjusted survival and mortality models presented in Section 3.5.2 (Table 6) are constrained by the small number of mortality events in this cohort (*n* = 11); adjustment was therefore restricted to a single clinicopathological covariate to preserve an adequate events-per-variable ratio, and the resulting confidence intervals are wide. These adjusted estimates should be regarded as exploratory and hypothesis-generating rather than definitive, and require confirmation in a larger, adequately powered, prospectively assembled cohort with a fully annotated Cox model incorporating multiple covariates simultaneously.

## 5 Conclusions

In conclusion, this multicenter study demonstrates that PD-L1 expression in breast cancer is more closely linked to histological grade than to pathological tumor stage or clinical disease extent, and that PD-L1 upregulation in tumor-infiltrating immune cells is mechanistically coupled to CD8+ cytotoxic T cell density a pattern consistent with adaptive immune resistance that is absent from the tumor cell PD-L1 compartment. CD8-high status defined by a ≥50% ratio threshold was more frequent in Luminal B than TNBC tumors, a finding that requires prospective validation with standardized thresholds. The association between CD8-high status and mortality persisted after adjustment for baseline distant metastasis, indicating that it is not solely attributable to more advanced disease at presentation, although this exploratory adjusted analysis was limited by the small number of mortality events. Rather than reflecting a causal adverse effect of cytotoxic infiltration, this association is most plausibly, though not definitively, consistent with exhaustion-driven immune dysfunction in a predominantly HR+ cohort — a state in which quantitatively abundant but functionally impaired CD8+ T cells co-occur with PD-L1 upregulation without conferring effective antitumor immunity. The predominance of the immunologically cold phenotype underscores the necessity of direct combined PD-L1/CD8 immune phenotyping for patient selection in checkpoint blockade strategies, as neither molecular subtype nor clinical stage reliably predicts immune phenotype. Prospective validation with event-annotated survival data, orthogonal transcriptomic profiling, and standardized CD8+ TIL ratio thresholds is required to establish the clinical utility of this combinatorial approach.

## Data Availability

The original contributions presented in the study are included in the article/supplementary material, further inquiries can be directed to the corresponding author.
